# The other side of the mark sheet: lessons learnt when medical students assess peers in formative clinical examinations

**DOI:** 10.3389/fmed.2024.1395466

**Published:** 2024-06-06

**Authors:** Helen Rienits

**Affiliations:** Graduate School of Medicine, Faculty of Science, Medicine and Health, University of Wollongong, Wollongong, NSW, Australia

**Keywords:** peer assessment, student, experience, formative, OSCE

## Abstract

This study aimed to investigate the experience of medical students assessing their cohort peers in formative clinical assessment. The exercise was designed to provide students with a formative experience prior to their summative assessment, and to determine what students could learn by being on the “other side of the mark sheet.” Students were grateful for the experience learning both from the assessment practice, and from the individual written feedback provided immediately afterwards. They also described how much they learnt from seeing the assessment from the assessor’s viewpoint, with many students commenting that they learnt more from being the “assessor” than from being the “student” in the process. Students were asked how they felt about being assessed by their peers, with some describing the experience as being more intimidating and stressful than when compared to assessment by clinicians. An interesting aspect of this study is that it also demonstrates some findings which suggest that the students’ current learning context appears to have an effect on their attitudes to their peers as assessors. It is possible the competitive cultural milieu of the teaching hospital environment may have a negative effect on medical student collegiality and peer support.

## Introduction

Peer assessors have been used by many medical training institutions to enable trainees to have a formative Objective Structured Clinical Examination (OSCE) experience at lower cost to the institution (1–4). Generally, when “peer” medical student assessors are used, they are in fact “near peer” and more advanced in their training compared to those they are assessing (2, 3). However, some recent studies have been undertaken using “same cohort” or “reciprocal” peers to ascertain the learning value in having these peers as assessors (4–6).

In addition to the opportunity to practice skills, a peer assessed formative OSCE can also provide students with individual feedback. Feedback in formative assessment has been shown to be a powerful aid to deep learning (7) and some excellent research has provided good advice re the process of giving this feedback (8), especially in relation to its timeliness, and individual descriptive text rather than just broad grades or marks. However, most of the studies conducted on the power of feedback in formative assessment have been done using experienced clinicians or professionals to provide the feedback to the trainee (7, 9). More studies are needed on the efficacy of feedback provided by peers, especially reciprocal peers.

OSCEs are widely used in health profession training to assess developing competence in clinical performance (10, 11). However, individual performance-based assessment can be a stressful experience for the trainee and performance-based anxiety has been shown to impact trainee performance (12, 13). Many medical schools therefore provide students with a formative OSCE experience in advance of the summative assessment to help them prepare, and relieve some of their anxiety.

The style of the mark sheet also provides its own challenges. Studies have noted that compared with using checklists, junior assessors struggle with the concept of global rating in mark sheets, even when there are good descriptions of the mark criteria (14, 15). When using student peer assessors, inter-assessor reliability of results has been found to be poor compared with that of experienced assessors (2, 3, 16, 17). However as the purpose of the formative OSCE is usually to provide the students with an opportunity to become familiar with the process and physical context of OSCE, inter-assessor reliability may not necessarily be considered a major objective.

Being assessed by peers in a formative OSCE enables the student to have an OSCE practice experience, and receive feedback on their performance. This should be a useful learning exercise for the student. However, there has been comparatively little research around the student experience of cohort peer assessment. Does being the assessor enable the student to become more clearly acquainted with the standards of knowledge and skills expected of their level of training? Does providing feedback for their peers provide the student with insight relating to how their own performance compares with the expected standard? Therefore this study aimed to investigate the student experience of assessing, and being assessed by, their cohort peers in a formative OSCE.

## Methods

This study aimed to investigate the research question through the student perspective. Students’ opinions were sought regarding how useful they found the exercise for their learning, both as a student and as an assessor. Both qualitative and quantitative data were collected in an effort to gauge the extent, and the depth, of responses to the questions.

During the compulsory formative OSCEs over 3 years: 2017 – 2019, medical students at the end of second (P1), third (P2), and fourth (P3) years, were assessed by their cohort peers, and had the opportunity to assess their peers. Students worked in groups of eight. During the first round, four of the students rotated around a circuit completing four different stations, while the other four students took the role of the assessor on one station each. In the second round, the students swapped roles and repeated the process with four new stations. Trained volunteers from the community played the roles of simulated patients. Location, clinical set up, OSCE timing and “bells,” and all station assessment materials, including the mark criteria and mark sheets, were in exactly the same format and standard as the summative OSCE for their respective Phase. Our OSCE mark sheets use global rating scales for each domain, with descriptive mark criteria of expected standards. The mark sheets also allow assessors to provide written feedback to the candidate, and our peer assessors were encouraged to complete this section as well when marking, so that all students received individual written feedback on their performance. Supervising clinical tutors also provided verbal feedback afterwards to each group of 8 students.

Immediately following the completion of the OSCE, each student collected their own mark sheets and viewed their written feedback. As this was a formative assessment, the school did not retain or record the grades or feedback. The students were then invited to complete an anonymous survey regarding the experience. Students had received prior information regarding the survey and were aware that their participation in the survey was voluntary.

The survey questions, specifically designed for this study, were based on a pilot study conducted in 2016 with P1 medical students. The results of the pilot are not included in this study, but it helped to refine the questions for this subsequent survey. Because the survey was conducted over 3 years, students who were first surveyed at the end of P1 (in 2017) were surveyed again at the end of P2 (in 2018), and then again at the end of P3 (in 2019). Other cohorts were similarly surveyed over sequential years. This allowed some longitudinal tracking of cohort responses as they progressed through the course. Individual student responses however, could not be tracked longitudinally due to the anonymous nature of the survey.

The context of the student training differs over the three Phases of our course P1, P2, and P3. P1 students spend most of their time studying the medical sciences, with the clinical component learnt during Clinical Skills on campus, and brief placements in local community practices. P2 students spend the Phase based in teaching hospitals rotating through standard blocks in Medicine, Surgery, Mental Health, etc. P3 students by contrast, spend the whole phase based in community practices, with added training in the Emergency Departments of local hospitals. Most P3 students are based in regional and rural centers for the year. Including students from all Phases enabled a comparison of cohorts across different levels of training and between different training contexts.

The survey aimed to ascertain what students could learn when they assess their peers in a formative OSCE. Survey questions were grouped under 4 topics to investigate their perception of the learning value of the exercise from:

(A) Participating in a formative OSCE both as a “student” (3), and as an assessor (2)
(B) Completing the mark sheet as an assessor (4)
(C) Being an assessor of their peers (3)
(D) Being assessed by their peers (6)

Students were asked to indicate their agreement with statements regarding these topics on a five-point Likert scale. Following each set of topic questions, students were asked to share “Any other comments?” in a free text box. The Human Research Ethics Committee of the university approved these studies - Ref Nos: 2017/030, 2018/010, & 2019/011.

## Results

There was a good response rate to the voluntary survey with 684 responses / 702 participants (97%) in total from P1, P2, and P3 students, during the 2017, 2018 and 2019 formative OSCEs.

Quantitative Results: The combined results for all three cohorts in all 3 years of the study are summarized in Table 1 and expressed as a rounded percentage of all those who answered that question.

**TABLE 1:** Student survey responses on their experience of the formative OSCE.

Table 1	SD	D	N	A	SA
**1A: On having this formative/practice OSCE**					
All OSCE practice is helpful to prepare for the summative OSCE	0	0	1	26	73
It was useful to practice with the bells and the timing	0	0	0	20	80
It helped to see the kind of scenarios and tasks for this phase	0	0	1	20	79
It helped to understand the standard expected for this phase	0	2	3	25	70
It helped to understand the marking process	0	0	4	24	72
**1B: On being an OSCE assessor**					
Global Judgmentis very difficult	3	23	25	41	8
I felt I needed more specific marking guidelines	5	23	25	26	6
It is difficult knowing how to split the borderline (pass/fail) grades	1	14	20	56	9
I learnt more being an “assessor” than being the “student”	2	13	35	37	13
**1C: On assessing my peers**					
It is difficult to be critical because we are assessing our peers	5	31	22	35	7
It is hard to mark your peers objectively	5	38	21	35	4
I do not feel competent to assess my peers	6	45	31	15	3
**1D: On being assessed by my peers**					
I didn’t mind being assessed by my peers at all	1	4	10	49	36
It is less stressful being assessed by my peers (than by clinicians)	3	18	29	32	18
It was intimidating being assessed by my peers	11	46	24	17	2
It felt less objective than being assessed by a clinician or a stranger	3	28	30	31	8
I do not feel my peers are competent to assess me	17	54	19	5	2
This exercise was not helpful because assessors were not real clinicians	25	49	16	8	2

The five-point Likert scale was graded as follows: SD, Strongly Disagree; D, Disagree; N, Neutral; A, Agree; SA, Strongly Agree.

(A) On participating in the formative OSCE. Student agreement with the statements in this section was over 95%. They agreed it was helpful to practice with the OSCE ‘bell’s, timing, and kinds of scenarios and tasks that they were likely to encounter in the summative as students. They also agreed (95%) that being an assessor helped them to understand the standard expected and how the marking process worked.
(B) On being an OSCE assessor. The next four statements (Table 1B) related to experiencing the OSCE effectively from “the other side of the mark sheet” and the challenges of OSCE assessment. Between 50 and 65% of students agreed with statements that using global judgmentand deciding the pass/fail borderline grades were difficult. When asked whether they learnt more from being the “assessor” than the “student” in the exercise, 50% of students agreed while 26% disagreed.
(C) On assessing my peers. The survey then covered the aspects of what it felt like to assess your peers (Table 1C). In almost all cases, the student assessors knew the student candidates they were assessing as friends and colleagues. This added a dimension to the process which many found difficult (42%).
(D) On being assessed by my peers. The final six statements (Table 1D) asked the students how they felt about being assessed by their peers. The majority of students did not mind being assessed by their peers in this formative setting (85% agreed). However, approximately 20% of students reported feeling that it was intimidating to be assessed by their peers.

Qualitative Results: Of the 684 responses to the survey, approximately 390 responses (57%) contained added comments in the free text boxes following the sets of quantitative questions. Selected examples representing frequently occurring themes in the comments are presented below under the relevant survey topic headings.

(A) On participating in the formative OSCE. There were many positive comments expressing how helpful students found the exercise and the feedback opportunities.

“*All up I enjoyed the formative- it will help me prepare for the summative and I now know what I need to work on*” (P1)

“*I appreciated the opportunity for feedback*” (P3)

“*Having a clinician around to give us advice when we are assessing was exceptionally helpful*” (P3)

(B) On being an OSCE assessor. There were many comments concerning having the opportunity, as “assessors,” to see the marking criteria and mark sheets as they applied to set scenarios and tasks.

“*Great to look at the marking criteria and get a feel about what assessors are looking for*” (P3)

“*To understand how the OSCE “worked” to assess a student’s capabilities.*” (P1)

“*Marking expectations were clear but the global judgment was most difficult*” (P2)

(C) On assessing my peers. Student comments reflected some of the issues they experienced when assessing peers.

“*Difficult removing preconceptions about your peers*” (P3)

“*I felt biased toward my friends over other colleagues and wanted to give them higher marks*” (P1).

“*It is hard to not prompt friends/peers when they are stuck*” (P1)

“*It’s hard giving negative feedback when they are your friends*” (P1)

There were also a number of comments about the experience of being able to watch a number of peers complete a task in their unique styles.

“*Great to see other student’s style of history and examination*” (P1).

(D) On being assessed by my peers. Most of the comments in this section came from the 20% of students who found assessment by their peers to be stressful or intimidating.

“*Sometimes it can be disheartening if you make errors in front of your peers but I trust their judgement*” (P1)

“*OK with some students but others are judgmental, unprofessional*” (P2)

Differences between the Phases in the peer- assessed OSCE.

**FIGURE 1:**
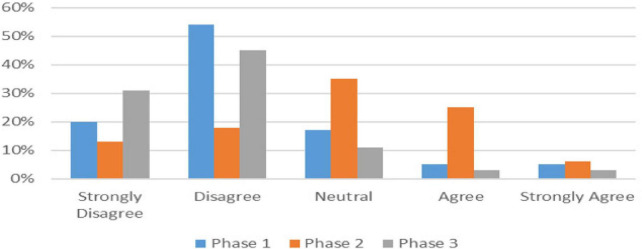
It felt less objective than being assessed by a clinician or a stranger.

**FIGURE 2:**
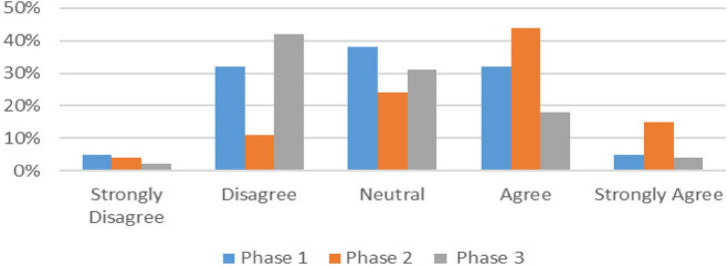
This was not helpful because the assessors weren’t real clinicians.

**FIGURE 3:**
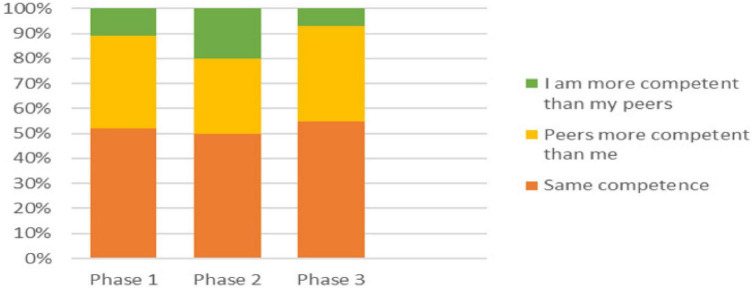
Differences in perceived competence to assess peers between the phases.

For the majority of questions, the student responses were very similar when compared across the three phases of training. There were however, a few questions where some differences were noted. These differences were between the phases (or stages of training) and were consistently noted across the 3 years of the study. The differences are displayed in the graphs in Figures 1–3. When asked how objective it felt being assessed by peers (Table 1D), there was a spread of responses which differed from Phase to Phase as illustrated in Figure 1. Phase 2 students differed to Phases 1 and 3.

The difference between the Phase 2 students and the other two phases was noted in another question relating to whether the students felt the formative OSCE was helpful despite being peer, rather than clinician assessed (Table 1D). Figure 2 shows Phase 2 mostly agreed that it was not helpful, while the other Phases mostly disagreed. The comments relating to this issue were entirely from Phase 2 students such as the following 2 examples.

“*It would be good to have clinicians so a more experienced person is marking rather than peers*”

“*Clinician feedback would be valuable as students don’t know enough to assess*”

Student perceptions regarding their competence to assess peers.

Finally, in one section of the survey, students were asked to indicate their level of agreement with the statement: “I do not feel competent to assess my peers” (Table 1C), and in the following section, they were asked to indicate their level of agreement with the statement: “I do not feel my peers are competent to assess me” (Table 1D). These two statements were tracked and compared individually for each student across 684 survey responses. Just over half the students indicated the same level of agreement to both statements (Figure 3).

Of those that differed, the majority felt that other students were more competent to assess them, than they were to assess others (range 30.5% - 38.5%). A small proportion however, felt they were more competent to assess their peers, than their peers were to assess them. Again, it was the Phase 2 students who differed from the other cohorts as depicted in Figure 3 (10% P1, 20% P2, and 7% P3). Using Chi Square with a Fisher’s Exact test, combining Phases 1 and 3, and then compared to Phase 2 with all three groups, and including the percentage of students who reported feeling the same level of competence as their peers, less competent than their peers, and more competent than their peers, *P* = 0.0465, confirming the difference in the Phase 2 response is statistically significant.

Almost all students who registered different “levels of competence to assess” between these two statements, only moved one “grade” of agreement one way or the other. However, in one section only, (the Phase 2 students who felt themselves more competent than their peers to assess others), 27% recorded a difference of two or more grades. These students believed that they were much more competent to assess their peers, than their peers were to assess them. This pattern was consistent across all the cohorts of students surveyed as they moved through from Phase 1, to Phase 2 and finally to the end of Phase 3.

## Discussion

As with most formative assessments, peer- assessed OSCEs are primarily intended as assessment for learning rather than an assessment of learning (18). Using the students to assess their peers was therefore not just a cost cutting exercise, but designed to enhance student learning. Being able to sit a practice OSCE in the format and at the standard of the summative assessment was in itself a learning opportunity. This research however was designed to investigate the student experience of assessing, and being assessed by, their cohort peers in a formative OSCE.

The survey indicated that our students felt they had learnt as much or more being the assessor than from being the student participant in the formative OSCE. Other studies have also noted that even “near peer” student assessors said they learnt much from being assessors of their junior colleagues in developing their own clinical skills (3). Some students noted it was very helpful to watch how different students tackle a station, and observe the different styles of communication. Other research has also noted that peer assessors found they had learnt a lot about communication skills when observing and assessing peers (4). However just seeing the written mark criteria and expected standards is in itself relatively superficial learning in the assessment of clinical competence (19).

Seeing the station from the assessor perspective and having to provide their peers with feedback can provide some insight into their own OSCE process and performance techniques. Others have shown that this kind of learning may change the way students both prepare for their summative assessments, and structure their performance (20, 21). It may result in improved OSCE marks, but again we must ask whether this truly represents deeper learning for future clinical practice, or just for assessment performance.

The survey results indicate that most students struggled with the difficulty of deciding global grades especially around the borderline mark. Clinician assessors also struggle being confident in this aspect of clinical assessment (22, 23), and it suggests that participation as an assessor in a formative OSCE can provide some insight into the essentially subjective process of performance-based assessment. Students also noted the difficulty marking friends objectively. The desire to help and encourage your friends rather than give “negative” feedback is similar to the problems clinician assessors experience when asked to assess their own student (24). The desire to help your colleague pass even if not at the standard expected, and the difficulty recognizing bias while struggling to be objective, are important self-reflective insights (25).

One of the unexpected findings from this study was the clear differences between the Phases in a small number of key areas. The student responses in terms of their perceived competence to assess relative to their peers, was clearly different in students in Phase 2 compared to the other phases. This same pattern was repeated in each of the 3 years that these studies were conducted so it was unlikely to be due to individual cohort variation. The major difference for students in Phase 2 is the training context that these students have been immersed in for the preceding year. In Phase 2 students have just completed a 12-month hospital placement rotating through various hospital disciplines. The teaching hospital is an intense and highly competitive atmosphere, and these junior medical students spend most of their placement time with junior doctors, who are themselves competing for more advanced specialist training positions.

As this survey was conducted at the end of the phase, it is possible that the “hidden curriculum” of deeply embedded cultural norms in the teaching hospital (26, 27), had an effect on the Phase 2 students’ self-perception of competence compared to their peers. Some of the responses seen in the Phase 2 students are at odds with the peer support, teamwork, collegiality, and compassion, which are professionalism skills we want our students to understand and develop (25). More recently, others in the field have shown that deeply embedded cultural norms, especially seen in the various contexts of medical training, can affect the way formative feedback is delivered (28), and student well-being, collegiality and compassion (26, 27). This is clearly an area that needs more attention and research.

This study aimed to investigate the student experience of assessing, and being assessed by, their cohort peers in a formative OSCE. Our peer assessed formative OSCEs were a feasible, and low-cost method, for giving students a formative experience to help prepare them for their summative OSCEs. Students had the opportunity to practice under formal exam conditions and processes, and many stated that they had learnt much from the opportunity of being in the role of the assessor themselves. As an “assessor,” students were forced to grapple with some deeper aspects of learning related to their developing professionalism skills. While difficult to quantitate, these skills are important in the long term for safe future clinical practice.

## Data availability statement

The original contributions presented in this study are included in the article/supplementary material, further inquiries can be directed to the corresponding author.

## Ethics statement

The studies involving humans were approved by the Human Research Ethics Committee of the University of Wollongong approved these studies - Ref No’s: 2017/030, 2018/010, & 2019/011. The studies were conducted in accordance with the local legislation and institutional requirements. The Ethics Committee/Institutional Review Board waived the requirement of written informed consent for participation from the participants or the participants’ legal guardians/next of kin because participants were informed ahead of time that their participation was voluntary and anonymous, and their completion of the survey was taken as tacit consent.

## Author contributions

HR: Conceptualization, Data curation, Formal analysis, Investigation, Methodology, Writing – original draft, Writing – review and editing.
